# Evaluating the diagnostic role of magnetic resonance imaging in trigeminal neuralgia, hemifacial spasm, and glossopharyngeal neuralgia: A controlled blinded study

**DOI:** 10.1016/j.bas.2025.105607

**Published:** 2025-09-25

**Authors:** Roberta Bonomo, Giulio Bonomo, Guglielmo Iess, Marco Paolo Schiariti, Jacopo Falco, Mario Stanziano, Francesco Restelli, Elio Mazzapicchi, Morgan Broggi, Ikrame Labiad, Francesco Acerbi, Giuseppe M.V. Barbagallo, Paolo Ferroli

**Affiliations:** a Department of Neurology, Fondazione IRCCS Istituto Neurologico C. Besta, Milan, Italy; b School of Medicine and Surgery, Kore University of Enna, Enna, Italy; c Department of Neurosurgery, Fondazione IRCCS Istituto Neurologico C. Besta, Milan, Italy; d Department of Neurological Surgery, Policlinico "G. Rodolico-S. Marco" University Hospital, Catania, Italy; e Neuroradiology Unit, Diagnostic and Technology Department, Fondazione IRCCS Istituto Neurologico C. Besta, Milan, Italy; f Neurosciences Department “Rita Levi Montalcini”, University of Turin, Turin, Italy; g Department of Translational Research and New Technologies in Medicine and Surgery, University of Pisa, Pisa, Italy; h Unit of Neurosurgery, University Hospital of Pisa, Pisa, Italy

**Keywords:** Diagnostic accuracy, Glossopharyngeal neuralgia, Hemifacial spasm, Magnetic resonance imaging, Neurovascular compression, Trigeminal neuralgia

## Abstract

**Introduction:**

Hyperactive dysfunction syndromes (HDS)—including trigeminal neuralgia, hemifacial spasm, and glossopharyngeal neuralgia—are typically associated with neurovascular compression (NVC) at the cranial nerve root entry/exit zone. MRI is widely employed to detect NVC, although its diagnostic reliability remains controversial, as NVC may also be observed in asymptomatic individuals, while in some symptomatic patients, radiological evidence can be absent.

**Research question:**

Can preoperative MRI reliably identify NVC in HDS patients undergoing microvascular decompression (MVD), and how does clinical information affect diagnostic accuracy?

**Materials and methods:**

We retrospectively analyzed preoperative 3 T MRIs from 42 HDS patients treated with MVD (2013–2023) and compared them with scans from age-matched healthy controls. Three experienced raters evaluated the images under five conditions with varying levels of clinical information, to assess how context influenced the detection of NVC.

**Results:**

Diagnostic accuracy increased in parallel with the amount of clinical information available. Under fully blinded conditions, correct identification of NVC remained below 50 %. Sensitivity improved when the affected nerve was disclosed (77.7 %) and further increased with full clinical context (83.3 %). Sensitivity decreased to 23.8 % when control scans were introduced without the raters’ awareness but rose to 88.9 % when controls were acknowledged. Interrater agreement remained low (k = 0.2–0.3) across all settings.

**Discussion and conclusion:**

MRI findings alone may be insufficient to confirm or exclude NVC in HDS. Interpretation should always be integrated with clinical data, and surgical decisions should not be based solely on imaging, particularly in symptomatic patients with negative scans.

## Highlights

• First study assessing MRI in all HDS using blinded raters and healthy controls
• MRI diagnostic accuracy improves when clinical information is available
• NVC may be absent on MRI in symptoms or present in asymptomatic individuals
• Blinded MRI assessment shows low interrater agreement in detecting NVC
• MRI alone is not sufficient to guide surgical indication in HDS patients

## 1 Introduction

Hyperactive dysfunction syndromes (HDS), namely trigeminal neuralgia (TN), hemifacial spasm (HS), and glossopharyngeal neuralgia (GPN), represent a spectrum of disorders characterized by an overexcitability of the cranial nerves caused by neurovascular compression (NVC) at the root entry/exit zone (REZ) (Jannetta, 1977; Bonomo et al., 2025). Complementary anatomical and hemodynamic factors, such as posterior fossa malformations and hypertension, contribute to HDS pathogenesis (Jannetta, 1967, 1980) Secondary mechanisms associated with local myelin remodeling and vascular pulsation lead to altered electrical impulse propagation (ephaptosis) along the cranial nerve roots, contributing to HDS manifestations (Haines et al., 1980; Hamlyn et al., 1992). Microvascular decompression (MVD) is typically performed by decompressing the relevant vessels along the REZ of the respective cranial nerves in subjects unresponsive to conservative treatment, with satisfactory success rates (Barker et al., 1995).

Magnetic resonance imaging (MRI) is the gold standard instrumental modality for identifying the NVC and excluding the potential presence of secondary causes of HDS, including multiple sclerosis, tumors, or vascular malformations (Seeburg et al., 2016). Nevertheless, some authors outlined the presence of the NVC as an occasional finding on neuroimaging investigation in patients not affected by HDS, suggesting that this finding is a necessary but not sufficient condition to develop HDS clinical symptomology. On the other hand, there is still insufficient evidence to support or deny the usefulness of MRI in identifying NVC (Gronseth et al., 2008).

Based on these premises, we aim to evaluate the predictive power of preoperative brain MRI in detecting the presence of a NVC in cases treated at our center for HDS through MVD surgery, through a blinded image analysis that includes a healthy control group.

To the best of our knowledge, this is the first study to collectively assess all major HDS to evaluate the ability of MRI to detect or rule out NCV, using a structured, multi-level, blinded questionnaire and a healthy control group.

## 2 Materials and Methods

Patients affected by HDS treated with MVD at our center, Fondazione IRCSS Istituto Neurologico Carlo Besta, Milan, Italy, were retrospectively collected from 2013 to 2023. An age-matched healthy control group was recruited. The study was performed following the ethical standards laid down in the 1964 Declaration of Helsinki and its later amendments. All patients were previously treated with conservative therapy. Detailed neurological and medical history, neurological examination of the cranial nerves, and motor and sensory functions were performed. All patients underwent standard brain contrast-enhanced MRI to rule out any secondary cause of symptomatic HDS. All of the subjects enrolled underwent a preoperative imaging protocol using a 3T scanner equipped with a 32‐channel coil (Achieva TX, Philips Healthcare BV, Best, NL), including the following sequences: 3D constructive interference in steady state (3D-CISS) [Philips equivalent: 3D-balanced-FFE]; 3-D time-of-flight magnetic resonance angiography (3D-TOF-MRA); 3-D T1-weighted sequencing with gadolinium (3D-T1-Gad) (Bonomo et al., 2022). MVD surgery was performed according to our protocol based on Janetta et al. (Ferroli et al., 2022a, 2022b)

Preoperative MRI brain images were then retrospectively reviewed by two expert neurosurgeons (PF and FJ) and one expert neuroradiologist (MS), who were asked to complete a questionnaire, consisting of five subsections, examining the following aspects: (1) Group A (totally blinded): to identify NVC side and involved nerve, while concealed about the clinical condition under study; (2) Group B (side-blinded): to identify NVC side, knowing the involved nerve; (3) Group C (not-blinded): to state whether there was evidence of NVC, non-blinded about the HDS (nerve and side) under study; (4) Group D (control-blinded): to state whether there was evidence of NCV, declared the HDS (nerve and side) under study, but blinded about the presence of controls in same cases; (5) Group E (control-not-blinded): to state whether there was evidence of NCV, declared the HDS (nerve and side) under study, non-blinded about the presence of controls in same cases.

### 2.1 Statistical analysis

Tests for statistical significance were analyzed in R version 4.2.1. A p-value <0.05 was considered statistically significant. We calculated sensitivity and specificity, Positive Predictive Value (PPV), and Negative Predictive Value (NPV) along with 95 % Confidence Intervals (95 % CI). We described quantitative variables as mean ± SD, and categorical variables as frequency (percent).

To evaluate the diagnostic performance of concealed raters in detecting NVC on preoperative MRI, a statistical analysis was conducted across five experimental conditions (Groups A–E), each corresponding to a different level of clinical information availability. The collected data included binary predictions (presence or absence of NVC) from three blinded raters on a cohort of patients undergoing MVD surgery.

For each Group-Rater combination, sensitivity, specificity, and accuracy were calculated, showing marked variability across conditions. Area Under the Curve (AUC) values were calculated for each Group-Rater combination.

#### 2.1.1 Mixed effects regression model

To account for the hierarchical structure of the data (observations nested within patients), a logistic mixed-effects regression model was applied. The model included “Group” and “Rater” as fixed effects, and “Patient” as a random effect. This allows the model to estimate how much of the variation in the probability of a positive diagnosis can be attributed to the experimental condition or the rater, while controlling for the fact that multiple evaluations may come from the same patient.

Technically, the model estimates the log-odds of a positive prediction (i.e., detecting NVC) as a linear combination of fixed effects (e.g., experimental group, rater identity) and a random intercept for each patient. This random intercept captures unobserved patient-specific variability, such as anatomical differences or symptom severity, that could influence the raters’ decisions independently of the experimental manipulations.

## 3 Results

Out of 42 HDS symptomatic patients, we enrolled 28 (66.6 %) female patients and 14 (33.3 %) male patients. Concerning the symptomatology, 17 (40.4 %) subjects presented with solely typical TN, 13 (30.9 %) patients with HS, seven (16.6 %) subjects with GPN, while five (11.9 %) subjects with combined TN and GPN. Intraoperatively, all HDS patients confirmed the NVC, of whom 18 (42.8 %) were on the right side and 24 (57.1 %) on the left one. There were no significant differences in time to recurrence, frequency of additional surgical interventions, or time to additional surgical interventions between study groups. The average duration of symptoms before surgery was 54 months (range, 6–168 months). At 3 months after MVD, relief of the symptomatology was complete, and all medication had been withdrawn in 39 (92.8 %) out of the 42 patients. Thirty-three percent of cases presented with adverse events during the first year of follow-up, including nausea and vertigo in 4 (9.5 %), hearing loss in 2 (4.7 %), ataxia in 2 (4.7 %), cerebrospinal fluid leakage in 1 (2.3 %), hyperemesis and dysarthria in 1 (2.3 %). No deaths were reported.

The specialists' interpretation of the first question in the questionnaire, regarding the NVC side and the involved nerve in preoperative MRI, while being unaware of the clinical condition (Group A: totally blinded), was accurate in less than 50 % of cases. However, when informed about the affected nerve (Group B: side-blinded), 77.7 % of the NCV side were properly diagnosed. In case of non-blindness about the NCV under study (Group C: not-blinded), raters correctly identified 83.3 % of cases. Conversely, when controls were introduced among the MRI under investigation (Group D: control-blinded), only 28.5 % of NCV cases were recognized, resulting in a sensitivity of 23.8 % and a specificity of 75 %. When raters were informed about the inclusion of control cases (Group E: control-not-blinded), they instead rose to 88.9 % and 58.3 %, respectively. Concerning MRI evaluation, the specialists showed low interrater agreement (k = 0.2) when concealed about the clinical condition, with a slightly higher concordance when acknowledged about the involved nerve and side (k = 0.3). The diagnostic accuracy in defining NVC diagnosis by specialists’ interpretation of pre-operative MRI imaging is shown in Table 1.

**Table 1:** Diagnostic accuracy of Questionnaire Items.

Diagnostic Accuracy by Raters	Group A		Group B		Group C		Group D		Group E	
	Actual Positive	Actual Negative	Actual Positive	Actual Negative	Actual Positive	Actual Negative	Actual Positive	Actual Negative	Actual Positive	Actual Negative
**Predicted Positive**	6 (TP)	0 (FP)	7 (TP)	0 (FP)	5 (TP)	0 (FP)	2 (TP)	1 (FP)	5 (TP)	2 (FP)
**Predicted Negative**	9 (FN)	0 (TN)	2 (FN)	0 (TN)	1 (FN)	0 (TN)	5 (FN)	3 (TN)	1 (FN)	2 (TN)

**Sensitivity%**	40 %		74.1 %		77.8 %		23.8 %		88.9 %	
**Specificity%**	0 %		0 %		0 %		75 %		58 %	
**PPV+**	100 %		100 %		100 %		62.5 %		76.2 %	
**NPV-**	0 %		0 %		0 %		36 %		77.8 %	

Group A: totally blinded; Group B: side-blinded; Group C: not-blinded; Group D: control-blinded; Group E: control-not-blinded.

FN: false negative; FP: false positive; NPV: negative predictive value; PPV: positive predictive value; TN: true negative; TP: true positive.

### 3.1 Descriptive accuracy metrics

Across all five experimental conditions (Groups A–E), descriptive analysis revealed substantial variability in diagnostic performance. Sensitivity was notably lower in conditions where clinical information was limited or absent (e.g., Group A), and significantly higher in Groups C and E, where full or declared clinical context was available. Specificity was generally more stable across groups but showed minor fluctuations due to the introduction of diagnostic distractors (e.g., in control-included conditions like Group D). Accuracy scores reflected the same trend, increasing in line with greater clinical information availability (Table 1).

### 3.2 Mixed effects model results

The logistic mixed effects regression model showed that both clinical context and evaluator identity significantly affected the likelihood of identifying NVC. Using Group A and RATER 1 as reference levels, the model showed that Groups C (β = 0.45, p < 0.00001) and E (β = 0.51, p < 0.00001) were associated with a significantly increased probability of detecting a NVC. This suggests that the availability of clinical context markedly enhances diagnostic performance. In contrast, Group B (β = 0.12, p = 0.23) and Group D (β = −0.10, p = 0.40) did not differ significantly from Group A, indicating that partial or ambiguous information had limited impact on the raters’ decisions.

Evaluator identity also played a significant role: RATER 2 showed a reduced tendency to identify NVC (β = −0.23, p = 0.011), while RATER 3 had a slightly higher propensity than RATER 1 to issue positive diagnoses (β = 0.16, p = 0.045). These findings highlight inter-rater variability and reinforce the need for integrated clinical-radiological evaluation frameworks.

### 3.3 ROC analysis

To further evaluate the diagnostic discrimination ability of each evaluator, AUC values were calculated for every Group-Rater combination. In Group A, where no clinical information was available, AUC values ranged from 0.59 to 0.63, indicating minimal discriminative ability just above chance. Group B, in which partial information was provided, yielded a slight improvement in diagnostic performance, with AUC values between 0.60 and 0.68. In contrast, Group C, characterized by full disclosure of clinical context, showed substantial enhancement in rater performance, with AUC scores ranging from 0.79 to 0.85. Group D, which introduced diagnostic distractors and blinded raters to the presence of controls, produced the weakest discriminative power, with AUC values between 0.54 and 0.57. Notably, Group E, where full clinical information was provided alongside clear identification of control cases, resulted in the highest AUC values across all raters, ranging from 0.81 to 0.88. These results reinforce the conclusion that diagnostic accuracy improves significantly with the availability and clarity of clinical context, consistent with both the descriptive and regression-based analyses.

## 4 Discussion and Conclusion

The results of our study collectively underscore the critical influence of clinical context and observer-dependent variability on the diagnostic accuracy of NVC using preoperative MRI.

Since its introduction in 2004 by Naraghi et al., MRI high resolution T2-weighted sequences by CISS have become the diagnostic imaging tool of choice for identifying the vascular conflicts around the cranial nerves, allowing the preoperative observation of the anatomical course of the causative vessels within the cisterns in the posterior fossa (Naraghi et al., 2004).

The comprehensive evaluation of MRI's role in diagnosing NVC in TN was examined across several studies. Initial studies, such as that by **Leal et al.,** evaluated the reliability of 3T MRI in detecting NVC in patients with TN, using blinded image analysis performed without knowledge of clinical symptoms or surgical findings. In their prospective series of 41 patients, MRI findings corresponded with intraoperative observations in most cases, with a reported sensitivity of 97.4 % and specificity of 100 %. Kappa coefficients indicated substantial agreement between imaging and surgical findings for identifying the responsible vessel (κ = 0.882), its location (κ = 0.813), and the site of compression (κ = 0.942). MRI also aligned with surgical grading of compression severity in most cases (Leal et al., 2011).

A review by the American Academy of Neurology (AAN) and the European Federation of Neurological Societies (EFNS) and their guidelines on TN management found that high-resolution MRI studies showed widely varying sensitivity (52–100 %) and specificity (29–93 %), mainly due to inconsistent imaging protocols. They concluded that there is insufficient evidence to support or deny the usefulness of MRI in identifying NVC, and it should be considered to identify patients with structural causes (Gronseth et al., 2008).

In the study by Tanrikulu et al., eight patients (seven with TN and one with vertigo) underwent high-resolution 1.5T MRI using T2 fast spin echo sequences. Image analysis was performed by evaluators who were aware of the patients’ clinical symptoms. MRI findings were compared with intraoperative observations, which were considered the diagnostic gold standard, resulting in a sensitivity of 100 % for detecting NVC (Tanrikulu et al., 2015). However, since mere neurovascular contact does not necessarily imply TN, the true diagnostic gold standard — as also adopted in our study — should be the presence of NVC observed intraoperatively combined with postoperative resolution of TN, thereby confirming a causal relationship between the NVC and clinical symptoms.

A large retrospective study of 1020 patients showed that, although preoperative MRI—particularly with high-resolution protocols—has a high positive predictive value (∼92 %) for detecting NVC in TN, its negative predictive value remains low (∼33 %) (Xu et al., 2022). Thus, the absence of radiological evidence of NVC is not sufficient to exclude the diagnosis, and clinical symptoms alone remain a key driver in the decision to proceed with surgical exploration. Nevertheless, it is important to note that in this study, image analysis was not performed in a blinded fashion: evaluators were aware of the patients’ clinical symptoms, and no healthy control group was included to assess diagnostic specificity—unlike in our study, which implemented blinded image review and included asymptomatic controls.

The study by Hitchon et al. confirms these findings, showing that high-resolution MRI has a high positive predictive value (95 %) but a low negative predictive value (27 %) in detecting NVC in TN (Hitchon et al., 2019). Unlike many earlier studies, this work employed a blinded evaluation of imaging, with reviewers unaware of clinical symptoms or surgical findings, thereby strengthening its methodological rigor. However, unlike our study, it did not assess whether the inclusion of healthy controls—creating the illusion of knowing the side of the conflict—could influence interpretation and introduce evaluative bias.

Antonini et al. conducted a blinded case-control study involving 25 patients with TN and 25 asymptomatic controls, using 3T MRI with high-resolution 3D T2-weighted CISS sequences to evaluate NVC at the REZ. Image analysis was performed independently by two neuroradiologists blinded to clinical data. The study found that the presence of NVC alone was common in both groups (80 % of symptomatic patients vs. 52 % of controls), resulting in moderate sensitivity (80 %) and limited specificity (48 %). However, when NVC was associated with morphological changes of the nerve—such as atrophy or distortion—the specificity and positive predictive value (PPV) reached 100 %, and interobserver agreement improved significantly (κ = 0.91) (Antonini et al., 2014). Consistently with our methodology, the study by Antonini et al. also incorporated a healthy control group, and their findings support the notion that NVC can be radiologically evident even in asymptomatic individuals. This highlights the importance of interpreting imaging findings within the clinical context.

A recent meta-analysis included studies on both TN and HS, evaluating the diagnostic performance of 3D multimodal image fusion (MIF) combining 3D TOF MRA with high-resolution T2-weighted imaging. Analyzing data from 390 patients across seven studies, the authors reported excellent diagnostic accuracy, with a pooled sensitivity of 97 %, specificity of 89 %, and an area under the ROC curve of 0.98 (Liang et al., 2023).

In a retrospective study, the authors analyzed high-resolution 3T MRI findings in 19 patients with GPN, focusing on NVC at the REZ of the glossopharyngeal nerve. Preoperative MRI successfully identified ipsilateral NVC in 18 of 19 cases, with the posterior inferior cerebellar artery (PICA) being the most common offending vessel. The compressive contact was most frequently observed at the proximal cisternal segment of the nerve, near the brainstem, where it is centrally myelinated. Surgical confirmation during MVD matched MRI findings in all operated cases, and most patients experienced complete or significant relief of symptoms (Gaul et al., 2011).

Although these studies collectively support the integral role of MRI in the preoperative assessment and planning for NVC syndromes, to date, there is no consensus on the implications of negative MRI in symptomatic HDS patients.

Our results highlight that the diagnostic interpretation of preoperative MRI is significantly enhanced by access to clinical context, suggesting that imaging alone may be insufficient for reliable identification of NVC. In particular, descriptive analyses demonstrated that sensitivity and accuracy improved substantially in conditions where raters had access to complete clinical information, while specificity remained more stable across conditions. The limited diagnostic performance observed in blinded settings (e.g., Group A and D) highlights the challenges of relying solely on imaging interpretation without clinical correlation. The application of a mixed effects logistic regression model confirmed that both the experimental condition and the identity of the rater significantly affected the likelihood of a positive diagnosis, emphasizing the presence of inter-rater variability. In particular, Groups C and E were associated with higher odds of accurate detection, and notable differences in decision tendencies were observed between raters. ROC analysis further reinforced these findings, with AUC values reaching their peak in the same groups, particularly when clinical data and control distinctions were made explicit. The findings demonstrate that while MRI can successfully detect NVC in the presence of strong clinical suspicion, its reliability significantly diminishes when negative controls are present. This implies that patients with negative MRIs may be at risk of misdiagnosis and may be left untreated due to potential false-negative interpretations of preoperative imaging.

MVD is a safe and effective solution in alleviating HDS, and the high rate of long-term benefit makes it an appealing treatment for eligible patients with medically intractable neuralgia (Barker et al., 1995).

Accordingly, the delayed treatment may indeed lead to worsening of symptoms and the occurrence of troublesome side effects of anti-inflammatory/anticonvulsant medications. Given its safety profile and long-term efficacy, MVD should remain a key treatment option in patients with persistent symptoms suggestive of HDS, even in the absence of radiological evidence of NVC.

In conclusion, we advocate for an integrated diagnostic workflow in HDS, in which MRI findings are interpreted in conjunction with detailed clinical assessment and, when appropriate, surgical exploration. This approach is particularly important in cases with negative imaging, where the risk of false negatives may lead to under-treatment.

## Consent to participate

Informed consent was obtained from all individual participants included in the study.

## Consent to publish

Patients signed informed consent regarding publishing their data.

## Ethical compliance statement

We confirm that we have read the Journal's position on issues involved in ethical publication and affirm that this work is consistent with those guidelines.

## Authors’ contribution

GB, GI, FR, and EM performed the clinical evaluation of all patients.MS, JF, and PF independently assessed the radiological images in a blinded fashion using the structured questionnaire. MPS, MB, and FA were responsible for performing the surgical procedures and perioperative management. IL collected and organized the dataset. RB, GB, and PF drafted the first version of the manuscript. All authors contributed to the interpretation of the results, critically revised the manuscript for important intellectual content, and approved the final version for submission.

## Data availability statement

The data that support the findings of this study are available from the corresponding author upon reasonable request.

## Funding

This work was supported by the Italian Ministry of Health (RRC).
